# Gait and balance performance of stroke survivors in South-Western Nigeria - A cross-sectional study

**DOI:** 10.11694/pamj.supp.2014.17.1.3001

**Published:** 2014-01-18

**Authors:** Adebimpe Olayinka Obembe, Matthew Olatokunbo Olaogun, Rufus Adedoyin

**Affiliations:** 1 Department of Medical Rehabilitation, College of Health Sciences, Obafemi Awolowo University, Ile-Ife, Osun State, Nigeria; 2 Department of Physiotherapy, Obafemi Awolowo University Teaching Hospitals Complex, Ile-Ife, Osun State, Nigeria

**Keywords:** Stroke, gait, balance, stroke survivors, rehabilitation, cadence, gait speed, Nigeria

## Abstract

**Introduction:**

Stroke survivors are often left with neurological and functional deficits, which impair their ability to walk and affect their balance. This study assessed gait parameters and balance performance among stroke survivors and examined the relationship between these two factors.

**Methods:**

Seventy stroke survivors (65.7% males) who were 6 months or more post stroke participated in this study. Using Observational Gait Analysis, the gait of participants was assessed by gait speed and cadence. Balance performance was assessed using the Activities-specific Balance Confidence scale for balance self-efficacy and Functional Reach Test for standing balance.

**Resuls:**

Participants had a mean age of 53.5±10.4 years. Forty five (64.3%) stroke survivors had haemorrhagic stroke while 25 (35.7%) had ischaemic stroke. The mean gait speed and cadence were 0.6±0.3m/s and 69.1±38.1 steps/min, respectively. The mean balance self-efficacy score was 66.5±17.7 and mean functional reach distance was 18.7±2.6cm. There were significant relationships between gait speed and balance self-efficacy (r =0.461, p =0.001) and between cadence and functional reach distance (r =0.247, p =0.020).

**Conclusion:**

This study concluded that stroke survivors with higher cadences had higher functional reach distances, and those with higher gait speeds had better balance self-efficacy. Gait speed and cadence are factors related to balance performance. These factors should be considered during gait and balance retraining and should go *pariparsu* in the rehabilitation of stroke survivors.

## Introduction

The most significant physical impact on stroke survivors is long term disability. Ambulation is a significant part of the functional recovery following stroke and this function depends on several factors including the size and location of the infarct and the premorbid health of the stroke survivor [1]. Dependence in mobility is one of the primary reasons of admission for inpatient rehabilitation after stroke. Much effort goes into helping these patients regain the ability to walk, at least in the home, prior to discharge. In spite of these efforts, approximately 35% of survivors with initial paralysis of the leg do not regain useful walking function, and 25% of all survivors are unable to walk without full physical assistance [2]. The result of this disability is a significant impact on the independence, quality of life and productivity of the survivors [3].

Gait is a major determinant of independent living. Therefore, it is not surprising that improvement of walking function is the most commonly stated priority of stroke survivors [4]. Approximately 80% of stroke survivors achieve this goal [5] though the quality of walking performance often limits endurance and quality of life [6]. A lot of time and resources are invested into rehabilitation to restore walking ability following stroke and reduce functional dependence.

Balance dysfunctions in stroke survivors are common and have significant impact on functional independence and overall recovery of the patient. Patients who have suffered a stroke, present with abnormal and delayed postural responses in the lower extremity muscles during standing displacements and distorted proprioception. They also demonstrate postural control problems such as loss of anticipatory activation during voluntary movements, increased sway during quiet standing, especially on the affected side, and decreased area of stability during weight shifting while standing. All these could result in clinical presentations such as loss of static and dynamic stability and reduced functional abilities [7].

Balance problems have been implicated in the poor recovery of activities of daily living (ADL) and mobility and an increased risk of falls [8]. Studies on balance impairments have shown that stroke survivors have greater postural sway than age-matched volunteers who are healthy [9–11]. They also have altered weight distribution patterns, so that less weight is taken through the weak leg, and they have smaller excursions when moving their weight around the base of support, especially in the direction of the weaker leg [12]. This pattern is seen in all aspects of balance—static, dynamic, or responses to external perturbations—and even in people with stroke with high levels of function, such as those who are ambulatory in the community.

Postural balance is closely related to gait ability [13]. A strong relationship has been reported between gait velocity and dynamic balance in the acute rehabilitation period among patients with first time ever stroke [14]. In a study of balance rehabilitation programs in which outcome measures consisted of gait velocity, timed stair climbing, self-assessment of ease of gait and balance under six sensory conditions, improvements in gait measures were correlated with improved balance [15]. Balance and gait impairments increase the risk of falls in older people [16]. During gait and balance retraining of stroke survivors, treatment goals are usually determined by analysing patients’ gait parameters and assessing the balance performance.

The incidence and prevalence of stroke have not been established in Nigeria. However, the frequency of stroke in hospital populations has varied from 0.9% to 4.0%, whereas among neurological admissions, stroke accounted for 0.5% to 45% of admissions [17]. There has been an increase in the incidence of stroke in Nigeria [18] and due to improved medical care many stroke victims now survive. Many patients will therefore need long term rehabilitation. Thus, assessing gait and balance is critical to stroke rehabilitation. Previous studies have separately reported gait [19–21] and balance performance [22, 23] in Nigerian stroke survivors, but the relationship between these attributeshas rarely been studied. This study therefore assessed gait parameters and balance performance of stroke survivors to investigate the relationship between them.

## Methods

### Study setting

This was a cross sectional study ofstroke survivors with hemiparesis attending outpatient physiotherapy clinics in the two teaching hospitals in Osun State, Nigeria. Using the sample size computation used by Eng [24] for descriptive clinical studies, we calculated a minimum sample size of 61 patients. Seventy stroke survivors were recruited fromObafemi Awolowo University Teaching Hospitals Complex (OAUTHC), Ile-Ife and Ilesa units; and Ladoke Akintola University of Technology Teaching Hospital (LAUTTH), Osogbo. Obafemi Awolowo University Teaching Hospitals Complex is one of the first generation teaching hospitals established by the federal government, while LAUTTH is jointly owned by the state governments of Osun and Oyo. These two tertiary hospitals are urban health centres that provide medical services and training for medical and other health students. By virtue of their locations and the scarcity of health care facilities in neighbouring areas, patients that are seen in these hospitals come from all areas of Osun State and south-western states in Nigeria -Oyo, Ondo, Ekiti, Kwara, Kogi, Lagos and Edo States.

### Participants

Fifty-two (74.3%) stroke survivors were recruited from OAUTHC and 18 (25.7%) from LAUTTH. The participants met the following criteria for inclusion in the study: a diagnosis of first episode of unilateral stroke by a neurologist; experienced stroke more than six months prior to the study; ability to understand and follow simple verbal instructions; ambulant before stroke; and ability to walk 10 metres in 1 minute or less without the physical assistance of a therapist or carer. This criterion corresponds to Functional Ambulation Categories (FAC) level 3 [25]. A patient was excluded if he or she had a history of other neurological pathology, conditions affecting balance (dementia, impaired conscious levels) and musculoskeletal conditions affecting the lower limbs. Stroke survivors who scored 0 to 2 on the FAC classification or who were dependent on a walker were also excluded from the study.

Twenty four (34.3%) women and 46 (65.7%) men participated in this study. Their ages ranged from 31-83 years with a mean of 53.5±10.4 years. Based on the heterogeneous nature of the study sample considering their ages and because most stroke survivors in Nigeria are between 50-70 years [17, 26], participants were classified into three age categories; less than 50 years, 50 to 70 years and more than 70 years.Twenty three (32.9%) participants had FAC of 3 (they could walk independently with supervision), while 43 (61.4%) had FAC of 4 (they could walk independently on level ground) and only 4 (5.7%) had FAC of 5 (they could walk independently).

### Ethical considerations

The protocol was approved by the Ethics and Research Committee of the Obafemi Awolowo University Teaching Hospitals Complex, Ile-Ife. All the participants received an explanation of the procedure of the study and gave informed consent prior to enrolment for assessment and data collection.

### Procedures

Demographic and clinical information were obtained directly from the participants and from their case records. From neurologists’ diagnosis which included radiological investigations, and our clinical findings, patients’ strokes were classified as haemorrhagic or ischaemic. Physical measurements were conducted in the gymnasia and walkways of the physiotherapy clinics of the selected hospitals on 15-metre walkways created on smooth floors for the purpose of the study.

The Functional Ambulation Categories test was used to assess walking ability by providing information on the level of physical support needed by patients to ambulate. Standing height and body weight of all the participants were measured using standardized protocols. Gait of participants were assessed with their gait speed and cadence by observational gait analysis (OGA). Balance performance was assessed using the Activities-specific Balance Confidence (ABC) scale and Functional Reach Test (FRT).

The gait parameters measured were gait speed and cadence. Measurements were taken by OGA. The measurement of gait speed was taken with the participant walking 10mwithout physical assistance while under the supervision of a physiotherapist. Distances of 2.5m were allowed before and after the 10m mark to allow for acceleration and deceleration respectively. Walking devices were allowed during the measurements. Five of the stroke survivors used quadripods during assessment.

Gait speed was assessed at comfortable self-paced walking speeds using a standard approach of observational gait analysis [27]. During each session, the participant walked 10m at a comfortable and at a self-paced walking speed. Timing with a digital stopwatch that registers time in seconds was manually initiated after the “go” instruction when the participant crossed the beginning of the 10m mark and stopped when the participant crossed the end of the 10m mark. Each participant rested for about one minute between each test [28]. Normal comfortable gait speed ranges from 1.3 to 2.5m/s [27]. Speed was calculated in metres per second by dividing the distance walked by the time required. This was recorded as the actual gait speed. Higher scores indicated faster gait speeds. To reduce measurement error of timed walking test, the mean of three repeated measurements was used.

Step length relates to body height and body height relates to gait speed in normal persons. Considering this relationship between height and gait speed and the established usefulness of height for reducing inter-individual variability in gait speed [29], gait speed was normalized by dividing speed by height. Height normalized speed (HNS) was determined by the formula;

Height-normalized speed = Actual speed (m/s)/Height (m) [27]

Cadence was recorded as the number of steps taken per minute. Higher scores indicated better cadence. Normal walking cadence is 90 - 120 steps/minute [30]. No encouragement to facilitate performance during walking session was permitted.

The Functional Reach Test (FRT) [31] was used to measure the maximum distance that the stroke survivors could reach forward horizontally beyond arm's length while maintaining a fixed base of support in standing with comfortable stance width [32]. Using a yardstick calibrated in centimetres (cm), mounted on the wall at shoulder height, each participant was asked to position the body close to, but not touching the wall with feet at a comfortable distance apart, the non-paretic arm outstretched and hand fisted. The starting position was noted by determining what number the metacarpophalangeal (MCP) joints lined up with on the ruler. Each participant was asked to reach as far forward as possible with the unaffected arm, without losing his or her balance or taking a step. The start and end measurements were recorded. The functional reach distance was the difference between the two measurements. Each participant was given two practice trials then the performance on an additional three trials was recorded and averaged. A 15 second rest break was allowed between trials. The participants were guided in case of loss of balance. The functional reach distance was recorded in centimetres (cm). Scores of 6-7 inches (15.2 - 17.8 cm) indicate a frail person with limited ability to perform ADLs and increased risk of falls [31]. Patients were classified as fallers (FRT distance < 17.8cm) and non-fallers (FRT distance > 17.8cm) using their FRT distances [33]. The use of assistive devices was not allowed during this test.

Perceived balance self-efficacy was assessed using theActivities-specific Balance Confidence (ABC) scale [34]. The Activities-specific Balance Scale which has been shown to be valid and reliable for people with stroke, is a self-efficacy scale that evaluates confidence in 16 functional activities, 9 of them outside the home [35]. Theratingsarebased on an 11-point scale ranging from 0% (“no confidence at all”) to 100% (“completely confident”). Participants were asked to rate their confidence that they will lose their balance or become unsteady in the course of completing 16 activities of daily living. The mean of the total score was recorded. A total score out of 100 was computed by taking the average of the item scores. The higher the ABC score, the higher the level of balance confidence. It took 10 - 20 minutes to administer the scale for each participant. Lajoie and Gallagher [36] reported that with an ABC Scale cut-off score of 67%, one can accurately classify people who fall 84% of the time. Patients in this study were classified as fallers (ABC score < 67%) and non-fallers (ABC score > 67%) using their ABC scale scores.

### Data Analysis

Data were analysed using both descriptive and inferential statistics. Descriptive statistics of mean, standard deviation, percentage, frequency, minimum and maximum values were determined for characteristics of the participants. Pearson product-moment correlation coefficient was used to determine the relationship between the gait parameters and balance performance. Independent samples t-test was used to determine the difference between fallers and non-fallers (risk of falls) and one way analysis of variance (ANOVA) was used to determine the difference among the age categories. Significance was set at 0.05 α-level. All statistical analyses were carried out using Statistical Package for Social Sciences (SPSS) 16.0 (SPSS Inc. Chicago, USA)

## Results

Table 1 shows the frequency values and percentages of the demographic (gender and age) and stroke (side of paresis, type of stroke and balance performance) characteristics of the stroke survivors. Twenty three (32.9%) stroke survivors had left-side paresis while 47 (67.1%) had right side paresis. Of the 70 stroke survivors, 45 (64.3%) had haemorrhagic type of stroke while 25 (35.7%) had ischaemic type.

**Table 1:** Characteristics of participants (N= 70)

Variable	Frequency	Percentage (%)
**Demographics**		
**Age categories (years)**		
**less than 50**	24	34.3
**50- 70**	44	62.9
**Above 70**	2	2.9
**Gender**		
**Female**	24	34.3
**Male**	46	65.7
**Stroke characteristics**		
**Side of paresis**		
**Left**	23	32.9
**Right**	47	67.1
**Type of stroke**		
**Haemorrhagic**	45	64.3
**Ischaemic**	25	35.7
**FAC Score**		
**3**	23	32.9
**4**	43	61.4
**5**	4	5.7
**ABC score category**		
**Fallers (£ 67)**	31	44.3
**Non fallers (> 67)**	39	55.7
**FRT distance category**		
**Fallers (£ 16 cm)**	27	38.6
**Non fallers (> 16 cm)**	43	61.4

FAC- Functional Ambulation Categories, FRT- Functional Reach Test, ABC- Activities-specific Balance Confidence, FAC 3 - Patient could walk independently with supervision, FAC 4 – Patient could walk independently on level ground, FAC 5 – Patient could walk independently.

The range, mean and standard deviation of the physical (weight and height) and stroke (stroke duration, gait and balance performance) characteristics of the participants are presented in Table 2. The stroke durations ranged from six months to twenty four months (mean = 18.3±8.8 months); Activities-specific Balance Confidence scale scores ranged from 21.4% to 97.9% (mean = 66.5±17.7%); functional reach test distances ranged from 7.6 cm to 39.4 cm (mean =18.7±4.6cm); gait speeds ranged from 0.11 m/s to 1.12 m/s (mean= 0.6±0.3m/s); and cadence ranged from 13.4 to 136.8 steps/minute (mean =69.1±38.1 steps/minute).

**Table 2:** Physical and stroke characteristics of participants

Variable (N = 70)	Minimum- maximum		Mean±SD
**Age (Years)**	31.0 -	83.0	53.5±10.4
**Height (m)**	1.7 -	1.9	1.7±0.8
**Weight (Kg)**	44.0 -	94.0	67.4±9.2
**Stroke duration (Months)**	6.0 -	24.0	18.3±8.8
**Body mass index (Kg/m**^**2**^**)**	16.8 -	33.7	24.3±2.8
**FAC score**	3.0 -	5.0	3.7±0.6
**ABC scale score (%)**	21.4 -	97.9	66.5±17.7
**FRT distance (cm)**	7.6 -	39.4	18.7±4.6
**Gait speed (m/s)**	0.1 -	1.1	0.6±0.3
**Height normalized speed**	0.1 -	0.7	0.3±0.2
**Cadence (steps/min)**	13.4 -	136.8	69.1±38.1

FAC- Functional Ambulation Categories, FRT- Functional Reach Test, ABC- Activities-specific Balance Confidence

The result of the Pearson product-moment correlation analysis showed a weak positive correlation between cadence and FRT distance (r = 0.247, p= 0.020); and a moderate positive correlation between gait speed and balance self-efficacy (r = .461 p = 0.001). But there was no significant relationship (p > 0.05) between FRT distance and balance self-efficacy (Table 3).

**Table 3:** Relationships between gait parameters and balance performance

Variable	ABC Scale score (r)	FRT distance (r)
**Gait speed (m/s)**	0.461**	0.115
**Height normalized speed**	0.069	0.107
**Cadence (steps/min)**	0.116	0.247*

* Correlation is significant at p<0.05

** Correlation is significant at p<0.01, ABC- Activities-specific Balance Confidence, FRT- Functional Reach Test.

There were significant differences in the FRT distance (p= 0.016) and ABC scores (p= 0.001) between fallers and non-fallers, when the stroke survivors were categorized according to risk of falls. However, there were no significant differences between fallers and non-fallers using both the ABC score and FRT distance categories in the following variables; gait speed, HNS and cadence. The result of the one way ANOVA showed no significant difference in gait speed (F= 0.230, p= 0.796), cadence (F = 0.442, p = 0.644), balance confidence (F = 1.137, p = 0.327) and functional reach distance (F= 0.338, p = 0.715) among the three age categories considered.

## Discussion

This study assessed the gait characteristics and the balance performance of stroke survivors and also explored the relationship between them. The gait speed observed was within the range reported by Olney and Richards [37] who observed that the average gait speed in stroke survivors ranged from 0.23m/s to0.73m/s depending on the severity of the hemiparesis. These mean values are below the values reported for normal subjects in self-paced walking [27]. Our results therefore demonstrate the impact of stroke on the ambulatory capability of stroke survivors. Although it is known that stroke survivors typically have reduced gait speed and cadence and present with abnormal and delayed postural responses, attempt to improve these parameters without compromising static and dynamic balances should be strongly considered in their walking re-education.

Mean ABC scale scores are similar to findings in previous studies of people living in the community after stroke [23, 38]. Ina Canadianstudy by Miller and Yiu [38] the mean balance confidence for the mostly male (71%) older adult sample (mean age 67.7±1 years) was 62±2. They found balance confidence to be an independent predictor of physical function, participation and stroke recovery. Their study provided support for Bandura′s premise that self-efficacy is more important than skill in predicting behaviour [39]. Balance confidence is a remedial condition; however, it is seldom addressed in rehabilitation [38]. The mean ABC score for stroke survivors in our study is lower than that of documented healthy community-dwelling elderly people [40]. The lower mean ABC Scale score for stroke survivors observed in our study indicates that stroke has a major effect on balance self-efficacy despite independent walking function.People with reduced balance confidence may thereforetry to avoid falls by limiting their participation in activities.

The risk of falls is high among stroke survivors and falling is one of the most frequent complications these patients present with in rehabilitation. According to Lamb et al [8], approximately 40% of people fall within the first year of a stroke. Falls therefore remain a common feature in the life of people with stroke after discharge from hospital. Lajoie and Gallagher [36] have suggested that with an ABC Scale cut-off score of 67%, people who fall 84% of the time can be accurately classified. Functional reach distance has also been associated with an increased risk of falls and frailty in elderly people who are unable to reach more than 15 cm [32]. We did not find significant differences in the gait speed, HNS and cadence between fallers and non-fallers, though the non-fallers had higher gait speed and cadence values. The reason for this result may be because all our participants could ambulate independently and therefore had similar gait speeds and balance performance.

The mean ABC score of stroke survivors in our study is also lower than that of stroke survivors in the study by Pang et al [41] among older adults with chronic stroke (>1 year). But higher than that of stroke patients in the study by Salbach et al [42] who included patients with more than one occurrence of stroke (11%) in their study. The higher scores obtained by Pang et al [41] probably occurred because all their participants were independent in walking. Whereas the lower mean score obtained in the study by Salbach et al [42] may be because a considerable proportion of people in their study required either supervision or physical assistance to walk. Salbach et al also included patients with less than 1 year post stroke and those with recurrent stroke, while our study included participants who were more than 6 months post stroke and did not include those with recurrent stroke. The difference in these results suggests that longer period of walking re-education (and balance retraining) of stroke survivors will be beneficial to their balance self-efficacy.

The moderate positive correlation between balance confidence and gait speed is similar to the findings in the study byBotner et al [43]. Guimaraes, and Issacs [44] also reported that gait speed is related to fall risk.This implies that stroke survivors with slower gait speeds have poorer balance confidence and vice versa. Findings from experimental studies have indicated that gait training enhances balance self-efficacy and that depression, age, sex, comorbidity, time post stroke, and functional mobility predict improvement in self-efficacy [42]. The findings in our study therefore suggest that slow gait speed is a potential risk factor for falls in chronic stroke survivors. Reports of previous studies have suggested that walking ability is a factor in falls because many reported cases of falls occurred during walking [45, 46]. Gait retraining will improve gait ability and balance self-efficacy and reduce the risk of falls in stroke survivors.

Themean FRT distance for the stroke survivors in this study is lower than that observed by Wolf et al [47] and Takatori et al [48].Many participants in the Wolf study used assistive devices (canes and ankle-foot orthoses), while none of the participants in our study used any assistive device to carry out this test. This implies that assistive devices can improve balance performance in stroke survivors by improving mobility and allowing for independence in the performance of mobility-related tasks. In the Takatori et al [48] studypatients were receivingintensive rehabilitation. The stroke survivors in our study were community-dwelling chronic stroke survivors who were not receiving intensive rehabilitation. Intensity of rehabilitation may enhance standing balance, thereby reducing the risk of falls in all categories of ambulatory stroke survivors. The evidence of the relationship between gait speed and balance performance has some implications for the rehabilitation of chronic stroke survivors. Reduced gait can negatively affect their balance ability and reduced balance may contribute to higher risk of falls. Ambulatory activities and balance retraining should be promoted during the rehabilitation of chronic stroke survivors.

Apart from a weak positive correlation with cadence, functional reach distance had no significant correlation with gait speed in our study similar to findings by Wolf et al [47], who found no significant relationship between gait speeds of stroke survivors assessed with a timed 10-metrewalk test and functional reach distance. Winstein et al [49] also found no association between gait function and standing balance. In evaluating the physical impairment and functional limitations of patients, it is clinically useful to assess walking capacity and monitor the recovery of gait performance. The findings of this study support the suggestions by van de Port et al [50] who argue thatthe ability to walk in the community requires more than gait speed alone. They showed in their study that improvement in balance control was the most important driver for improvement in hemiplegic gait. Balance control is therefore an important independent compensatory factor enabling patients to walk in the community despite lower gait speeds, suggesting that patients with a slow walking speed seem to be able to compensate by an appropriate use of walking aids and sufficient control of balance walker.

There are several study limitations that warrant acknowledgment. First, because the stroke survivors in our study could ambulate independently, the findings of this study may not be generalized to stroke survivors who cannot do so. Second, we used convenience sampling and therefore findings may not be generalizeable. Further research with a more representative sample is therefore necessary to explore other factors that may affect gait and balance in Nigerian stroke survivors with varying degrees of recovery.

## Conclusion

Stroke survivors with higher cadences had higher functional reach distances, and those with higher gait speeds had better balance self-efficacy. This implies that gait speed and cadence are factors related to balance performance and should be considered during balance and gait retraining and should go pari per su in the rehabilitation of stroke survivors.Rehabilitation should focus not only on improving gait speed and balance performance, but also on other factors that are conditional for becoming an independent community.

## References

[1] Herbert R, Moore S, Moseley A, Schurr K, Wales A (1993). Making inferences about muscle forces from clinical observations. Australian Journal of Physiotherapy..

[2] Hendricks HT, van Limbeek J, Geurts AC, Zwarts MJ (2002). Motor recovery after stroke: a systematic review of the literature. Arch Phys Med Rehabil..

[3] Rosamond W, Flegal K, Furio K (2007). Heart Disease and Stroke Statistics-2007 update: A Report From the American Heart Association Statistics Committee and Stroke Statistics Subcommittee. Circulation..

[4] Bohannon RW, Horton MG, Wikholm JB (1991). Importance of four variables of walking to patients with stroke. Int J Rehabil Res..

[5] Wade DT, Hewer RL (1987). Functional abilities after stroke: Measurement natural history and prognosis. J NeurolNeurosurg Psychiatry..

[6] Maulucci R, Eckhouse R (2011). A real-time auditory feedback system for retraining gait. ConfProc IEEE Eng Med Biol Soc.

[7] Hamzat TK, Fashoyin OF (2007). Balance retraining in post stroke patients using a simple, effective and affordable technique. Afr. J. Neurol. Sci..

[8] Lamb SE, Ferrucci L, Volapto S, Fried LP, Guralnik JM (2003). Risk Factors for Falling in Home-Dwelling Older Women with Stroke. The Women's Health and Aging Study. Stroke..

[9] Lamontagne A, Paquet N, Fung J (2003). Postural adjustments to voluntary head motions during standing are modified following stroke. ClinBiomech..

[10] Corriveau H, Hébert R, Raîche M, Prince F (2004). Evaluation of postural stability in the elderly with stroke. Arch Phys Med Rehabil..

[11] deHaart M, Geurts AC, Huidekoper SC, Fasotti L, van Limbeek J (2004). Recovery of standing balance in post-acute stroke patients: a rehabilitation cohort study. Arch Phys Med Rehabil..

[12] Tyson SF, Hanley M, Chillala J, Selley A, Tallis RC (2006). Balance disability after stroke. PhysTher..

[13] Nichols DS, Miller L, Colby LA, Pease WS (1996). Sitting balance: its relation to function in individuals with hemiparesis. Arch Phys Med Rehabil..

[14] Langhammer B, Lindmark B, Stanghelle JK (2006). The relation between gait velocity, static and dynamic balance in the early rehabilitation of patients with acute stroke. Advances in Physiotherapy.

[15] Bonan IV, Yelnik AP, Colle FM (2004). Reliance on visual information after stroke. Part II: Effectiveness of a balance rehabilitation program with visual cue deprivation after stroke: a randomized controlled trial. Arch Phys Med Rehabil..

[16] Melzer I, Benjuya N, Kaplanski J (2004). Postural stability in the elderly: a comparisonbetween fallers and non-fallers. Age Ageing..

[17] Ogun SA, Ojini FI, Ogungbo B, Kolapo KO, Danesi MA (2005). Stroke in South West Nigeria: A 10-Year Review. Stroke..

[18] Obiako OR, Oparah SK, Ogunniyi A (2011). Prognosis and outcome of acute stroke in the University College Hospital Ibadan, Nigeria. Niger J ClinPract..

[19] Olawale OA, Akinfeleye AM (2002). Effects of strengthening of lower limb muscle groups on some gait parameters in adult patients with Stroke. Journal of Nigeria Society of Physiotherapy..

[20] Obembe AO, Olaogun MO, Adedoyin RA (2010). Gait Characteristics of Hemiparetic Stroke Survivors in Osun, Nigeria, African Journal for Physical, Health Education. Recreation and Dance..

[21] Onigbinde AT, Mustapha MK (2010). Effect of six weeks cycle ergometry on selected Gait Parameters of Stroke Survivors. http://ijahsp.nova.edu/articles/Vol8Num3/onigbinde.htm.

[22] Obembe AO, Olaogun MO, Adedoyin RA, Lamidi RE (2011). Determinants of Balance Performance in Hemiparetic Stroke Survivors. Turkish Journal of Physical Medicine and Rehabilitation..

[23] Olaogun MOB, Lamidi RE, Obembe AO (2011). Balance Confidence and Standing Balance Performance among Stroke Survivors with Hemiparesis. J. Med. Med. Sci..

[24] Eng J (2003). Sample Size Estimation: How Many Individuals Should Be Studied?. Radiology..

[25] Holden MK, Gill KM, Magliozzi MR, Nathan J, Piehl-Baker L (1984). Clinical gait assessment in the neurologically impaired. Reliability and Meaningfulness. PhysTher..

[26] Owolabi MO, Ogunniyi A (2009). Profile of health-related quality of life in Nigerian stroke survivors. European Journal of Neurology..

[27] Bohannon RW (1997). Comfortable and maximum walking speed of adults aged 20-79 years: reference values and determinants. Age Ageing..

[28] Kollen B, Kwakkel G, Lindeman E (2006). Time dependency of walking classification in stroke. PhysTher..

[29] Al-Obaidi S, Wall JC, Al-Yaqoub A, Al-Ghanim M (2003). Basic gait parameters: A comparison of reference data for normal subjects 20 to 29 years of age from Kuwait and Scandinavia. JRRD..

[30] Murray MP, Drought AB, Kory R (1964). Walking patterns in normal men. The Journal of Bone and Joint Surgery..

[31] Duncan PW, Weiner DK, Chandler J, Studenski S (1990). Functional reach: a new clinical measure of balance. J Gerontol..

[32] Jonsson E, Henriksson M, Hirschfeld H (2002). Does the Functional Reach Test Reflect Stability Limits In Elderly People?. J Rehabil Med..

[33] Duncan PW, Studenski S, Chandler J, Prescott B (1992). Functional reach: predictive validity in a sample of elderly male veterans. J Gerontol..

[34] Powell LE, Myers AM (1995). The Activities-Specific Balance Confidence (ABC) Scale. J Gerontol A BiolSci Med Sci..

[35] Lord SE, Rochester L (2005). Measurement of Community Ambulation after Stroke: Current status and future developments. Stroke..

[36] Lajoie Y, Gallagher SP (2004). Predicting falls within the elderly community: comparison of postural sway, reaction time, the Berg balance scale and the Activities-specific Balance Confidence (ABC) scale for comparing fallers and non-fallers. Arch of Gerontol and Geriatr..

[37] Olney SJ, Richards C (1996). Hemiparetic gait following stroke. Part I: Characteristics. Gait Posture..

[38] Miller W, Yiu J (2010). Balance Confidence Predicts Physical Function, Participation, and Stroke Recovery after Inpatient Rehabilitation. https://www.wfot.org/wfot2010/program/pdf/1838.pdf.

[39] Bandura A (1982). Self-efficacy mechanism in human agency. Am Psychol..

[40] Hatch J, Gill-Body KM, Portney LG (2003). Determinants of balance confidence in community-dwelling elderly people. PhysTher..

[41] Pang MY, Eng JJ, Miller WC (2007). Determinants of Satisfaction with Community Reintegration in Older Adults with Chronic Stroke: Role of Balance Self-Efficacy. PhysTher..

[42] Salbach NM, Mayo NE, Robichaud-Ekstrand. S, Hanley JA, Richards CL, Wood-Dauphinee S (2006). Balance self-efficacy and its relevance to physical function and perceived health status after stroke. Arch Phys Med Rehabil..

[43] Botner EM, Miller WC, Eng JJ (2005). Measurement properties of the Activities-specific Balance Confidence Scale among individuals with stroke. DisabilRehabil..

[44] Guimaraes RM, Isaacs B (1980). Characteristics of the gait in old people who fall. IntRehabil Med..

[45] Hyndman D, Ashburn A, Stack E (2002). Fall events among people with stroke living in the community: circumstances of falls and characteristics of fallers. Arch Phys Med Rehabil..

[46] Hyndman D, Ashburn A (2003). People with stroke living in the community: attention deficits, balance, ADL ability, and falls. DisabilRehabil..

[47] Wolf SL, Catlin PA, Gage K, Gurucharri K, Robertson R, Stephen K (1999). Establishingthe reliability and validity of measurements of walking time using theEmory Functional Ambulation Profile. PhysTher..

[48] Takatori K, Okada Y, Shomoto K, Shimada T (2009). Does assessing error in perceiving postural limits by testing functional reach predict likelihood of falls in hospitalized stroke patients?. ClinRehabil..

[49] Winstein CJ, Gardner ER, McNeal DR, Barto PS, Nicholson DE (1989). Standing balance training: effect on balance and locomotion in hemiparetic adults. Arch. Phys. Med. and Rehab..

[50] van de Port IG, Kwakkel G, Lindeman E (2008). Ambulation in Patients with Chronic Stroke: How is it related to gait speed?. J Rehabil Med..

